# Evidence for regulated expression of Telomeric Repeat-containing RNAs (TERRA) in parasitic trypanosomatids

**DOI:** 10.1590/0074-02760170054

**Published:** 2017-08

**Authors:** Jeziel D Damasceno, Gabriel LA Silva, Christian Tschudi, Luiz RO Tosi

**Affiliations:** 1 Universidade de São Paulo, Faculdade de Medicina de Ribeirão Preto, Departamento de Biologia Celular e Molecular e Bioagentes Patogênicos, Ribeirão Preto, SP, Brasil; 2 Yale UniversitySchool of Public Health, Department of Epidemiology of Microbial Diseases, New Haven, CT, USA

**Keywords:** TERRA, *Trypanosoma brucei* telomere, *Leishmania* telomere, telomere transcription

## Abstract

The Telomeric Repeat-containing RNAs (TERRA) participate in the homeostasis of telomeres in higher eukaryotes. Here, we investigated the expression of TERRA in *Leishmania spp*. and *Trypanosoma brucei* and found evidences for its expression as a specific RNA class. The trypanosomatid TERRA are heterogeneous in size and partially polyadenylated. The levels of TERRA transcripts appear to be modulated through the life cycle in both trypanosomatids investigated, suggesting that TERRA play a stage-specific role in the life cycle of these early-branching eukaryotes.

Telomeres of yeast and mammalian chromosomes are transcribed into long non-coding RNAs, known as Telomeric Repeat-containing RNAs (TERRA) (Azzalin et al. 2007). In mammals, RNA polymerase II-driven transcription of TERRA proceeds from within subtelomeric regions towards the chromosome ends and at least a proportion of TERRA molecules contains a 5’-end 7-methylguanosine cap and a 3’-end poly(A) tail (Porro et al. 2010). Recent advances in the understanding of TERRA function has indicated its participation in telomere homeostasis by modulating telomerase activity, recruiting chromatin modifying factors and also by mediating the capping of chromosome ends (Azzalin & Lingner 2015). Similar to other eukaryotes, the linear chromosomes of trypanosomatids are capped by telomeres composed of TTAGGG repeats and participate not only in protection of chromosome ends, but also in the control of expression of subtelomeric genes (El-Sayed et al. 2005, Glover et al. 2007). Subtelomeric and telomeric regions are pivotal for antigenic variation in *Trypanosoma brucei*, a process that allows evasion from the host immune system during infection (Li 2015). Despite the growing body of information on trypanosomatid telomere biology (Janzen et al. 2004, Jehi et al. 2014, Devlin et al. 2016), our knowledge of TERRA transcription and function in these early-branching eukaryotes remains limited. Here, we report the detection of TERRA transcripts in *Leishmania spp*. and *T. brucei*; analyse the structure of the 5’ and 3’ ends of TERRA molecules; and present evidence for modulated TERRA expression through the life cycle of these trypanosomatids.

*Detection of TERRA in Leishmania* - To investigate the potential transcription of telomere repeats in different *Leishmania* species, Northern blots of total RNA were initially probed with the oligonucleotides (CCCTAA)_15_ and (TTAGGG)_15_ that allow the distinction between sense (UUAGGG)_n_ and antisense (CCCUAA)_n_ transcripts, respectively (Fig. 1A). The sense transcripts were detected as a smear of hybridisation between 0.5 and 10.0 Kb revealing the presence of TERRA RNAs in these *Leishmania* species. In contrast, antisense transcripts were not detectable under the same conditions. These findings are in agreement with the work of Rudenko and Van der Ploeg (1989), who found that the transcription of telomeres in different kinetoplastid species was predominantly unidirectional and proceeded from a chromosomal internal region towards the chromosomal end.

**Fig. 1:**
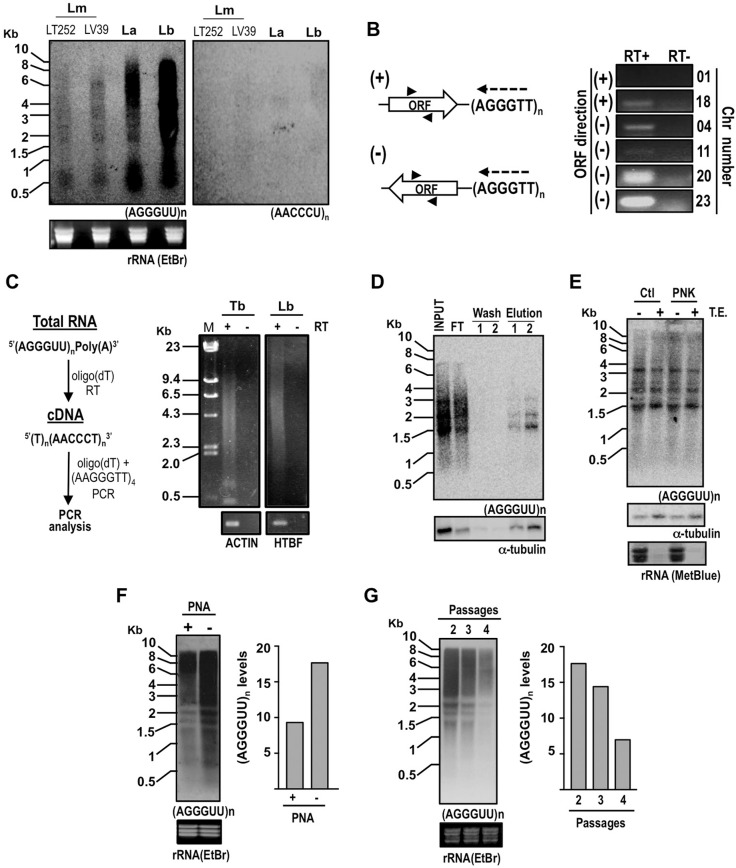
: transcription of UUAGGGn-containing RNAs (TERRA) from *Leishmania* telomeres. All the experiments in the figure were performed using RNA from promastigote forms. (A) Total RNA (~30 µg) from *L. major* (Lm; cell lines LT252 and LV39), *L. amazonensis* (La) and *L. braziliensis* (Lb) was subjected to Northern blotting and probed with 32P-end labelled (CCCTAA)15 or (TTAGGG)15 oligonucleotides. rRNA ethidium bromide (EtBr) indicates ribosomal RNAs stained with EtBr before transferring to the membrane and serves as loading and quality control of total RNA. (B) Reverse transcription polymerase chain reaction (RT-PCR) was performed to detect TERRA derived from different *L. major* telomeres; left panel, the diagram indicates the strategy used: the reverse transcription step (RT) was performed with (CCCTAA)15 oligonucleotide (dashed arrow); the cDNA generated was used in a PCR reaction performed with oligonucleotides (arrowheads) specific for the ORF (white arrow) closest to the telomere ((TTAGGG)n); ORFs orientated from the internal portion of the chromosome towards telomeres are indicated as (+); ORFs orientated from the telomeres towards the internal region of the chromosome are indicated as (-); right panel shows EtBr-staining of PCR products after fractionation in agarose gels; the cDNAs used as template for the PCR were generated in reverse transcription reactions performed in the presence (RT+) or absence (RT-) of Reverse Transcriptase; the specific primers for the PCR were designed for ORFs LmjF01.0010, LmjF18.0010, LmjF04.0010, LmjF11.0010, LmjF20.1620 and LmjF23.0030. (C) Left scheme indicates the approach for detection of TERRA by RT-PCR; total RNA (3 µg) from *Trypanosoma brucei* (Tb; bloodstream form, SM cell line) and *L. braziliensis* (Lb) was treated with DNAseI and submitted to reverse transcription reaction using oligo(dT) as primer; RT+ and RT- indicates addition or omission of reverse transcriptase enzyme from the cDNA synthesis reaction; the cDNA generated was used as template in a PCR reaction with oligo(dT) and a specific telomere sequence (AGGGTT)4 as primers; right panel shows Ethidium Bromide-staining of PCR products; in the bottom, actin and HTBF genes were used as positive controls for polyadenylated RNA in *T. brucei* and *L. braziliensis*, respectively. (D) Total RNA from *L. braziliensis* was fractionated on an oligo(dT) resin (Oligotex mRNA Mini Kit, QIAGEN); equivalent volumes from Input, flow through (FT), washes and eluted material were analysed by Northern blotting as in (A); samples were probed for a-tubulin as a positive control for the binding of poly(A)+ RNA. (E) Total RNA (~15 µg) from *L. braziliensis* was left untreated (Ctl) or subjected to polynucleotide kinase treatment (PNK) before incubation with terminator exonuclease (TE); samples were subjected to Northern blot analysis as in (A) and probed for a-tubulin as a control for 5’ CAP-containing RNA; rRNA (MetBlue) refers to rRNA staining with methylene blue after transfer to the membrane and serves as control for 5’mono-phosphorylated RNA. (F) Metacyclic and procyclic parasites were purified as previously described (Sacks et al. 1985). Briefly, stationary-phase *L. major* (LV39) promastigotes were incubated with the lectin PNA and agglutinated cells (PNA+) were separated from metacyclic parasites (PNA-) by centrifugation; the purification yield was 2.0 to 3.5%; the typical metacyclic morphology was used to confirm the sample purity; total RNA (~50 µg) from PNA+ and PNA- cells were subjected to Northern blot analysis as in (A); TERRA signal was quantified with ImageJ software, normalised with rRNA and plotted on the graph on the right; similar results were observed in another independent experiment. (G) Total RNA (~50 µg) extracted from *L. major* (LV39) at the indicated number of passages after isolation from mouse lesions, were subjected to Northern blot analysis as in (A); graph on the right shows quantification of TERRA signal as in (F); similar results were observed in three independent experiments.

To investigate whether the unidirectional TERRA transcript resulted from readthrough transcription of subtelomeric-located genes, we used reverse transcription-polymerase chain reaction (RT-PCR) to analyse the transcription at specific telomere ends of *Leishmania major* (Fig. 1B). The reverse transcription reaction used the telomere repeat oligonucleotide and the PCR step used specific primers for the first ORF upstream of the telomere. The chromosome ends analysed differed in the direction of the transcription unit upstream of the telomeric end. Thus, in the left arm of chromosomes 01 and 18 the transcription unit starts several kilobases upstream and proceeds towards the telomere, while in the left arm of chromosomes 04, 11, 23 and in the right arm of chromosome 20 the transcription unit starts within subtelomeric regions and proceeds away from the telomere. Thus, if TERRA is a by-product of polycistronic transcription, it should be detected only from telomeres downstream of ORFs transcribed towards the chromosome end. Surprisingly, RT-PCR revealed that TERRA was detected in the chromosomes with transcription units directed away from telomeres (Fig. 1B), which would argue against the idea of TERRA solely being the product of readthrough transcription downstream of telomeric genes. The presence of a transcription start site within the subtelomeres of *Leishmania* chromosomes 04, 11, 20 and 23 could mediate the transcription of TERRA in the other direction. In support of this hypothesis, the mapping of acetylated histone H3 loci, which are believed to mark transcription initiation regions along *Leishmania* chromosomes, identified H3ac-enriched loci within the subtelomeres of chromosomes 04, 11, 20 and 23 (Thomas et al. 2009).

Next, we investigated whether TERRA transcripts from *L. braziliensis* are polyadenylated. By using RT-PCR analysis we were able to detect TERRA transcripts in cDNA that was synthetised using oligo(dT) as primer (Fig. 1C). Moreover, affinity chromatography using an oligo(dT) resin revealed that a small proportion of TERRA molecules was eluted in the poly(A)+ fraction, indicating that at least a subset of the total TERRA is polyadenylated in *L. braziliensis* (Fig. 1D). This finding is similar to the observation that approximately 10% of the TERRA transcripts are polyadenylated in human cells and in *Schizosaccharomyces pombe* (Porro et al. 2010, Bah et al. 2012). It is noteworthy that TERRA species of ~1.6, ~2.1 and ~3.1 Kb were enriched in the poly(A)+ fraction (Fig. 1D). Although speculative, it is possible that the relative higher abundance of these particular species in both total and poly(A)+ RNA fractions, can be the result of differential transcription rates of specific telomeres or differential stabilisation of particular TERRA molecules by RNA-binding factors. To gain insight into the 5’-end structure of *Leishmania* TERRA, we tested the resistance of this RNA to Terminator Exonuclease (TE) treatment, which preferentially degrades 5’-mono-phosphorylated RNA. Northern blot analysis of total RNA did not reveal significant decrease in TERRA signal upon TE treatment (Fig. 1E), suggesting that TERRA 5’-ends are not mono-phosphorylated. Prior treatment of total RNA with T4 Polynucleotide Kinase (PNK), which catalyses the phosphorylation of 5’-hydroxyl ends, did not seem to interfere with the susceptibility of TERRA molecules to TE treatment. As expected, a-tubulin mRNA was resistant to TE treatment while rRNA was almost completely degraded by the exonuclease treatment. Altogether, these analyses suggested that TERRA transcripts do not have a free 5’-hydroxyl or a 5’-mono-phosphorylated end, but further investigations are required to determine if *L. braziliensis* TERRA contains a 5’-end 7-methylguanosine cap.

To gain insight into the expression of TERRA at specific stages of the *Leishmania* life cycle, we compared TERRA levels in procyclic and metacyclic parasites. We used the lectin peanut agglutinin (PNA) protocol to purify metacyclic parasites from stationary-phase promastigotes of *L. major* (Sacks et al. 1985). We then performed Northern blot analysis with RNA prepared from procyclic stationary phase parasites (PNA+) and metacyclic parasites (PNA-) and observed that the levels of TERRA transcripts were elevated in the RNA samples from PNA- parasites (Fig. 1F). Next, we examined TERRA levels in parasites isolated from mouse lesions after a number of passages. As shown in the Northern blot analysis and also in the quantification in Fig. 1G, the levels of TERRA were reduced after subsequent passages of the parasite as promastigotes in culture. These observations suggested that TERRA levels could be modulated during the establishment and/or maintenance of *Leishmania* infection.

*Detection of TERRA in T. brucei* - To test if differential expression of TERRA through development is restricted to *Leishmania*, we compared the levels of TERRA in RNA samples obtained from *T. brucei* bloodstream (BS) and procyclic (PC) forms (Fig. 2A). Similar to *Leishmania* and as previously reported (Rudenko & Van der Ploeg 1989), the TERRA transcripts were detected as a smear between 0.5 and 10.0 Kb in both BS and PC forms. Notably, TERRA levels were substantially higher in BS forms when compared to PC forms, suggesting that *T. brucei* TERRA is differentially expressed between these two life cycle stages.

**Fig. 2:**
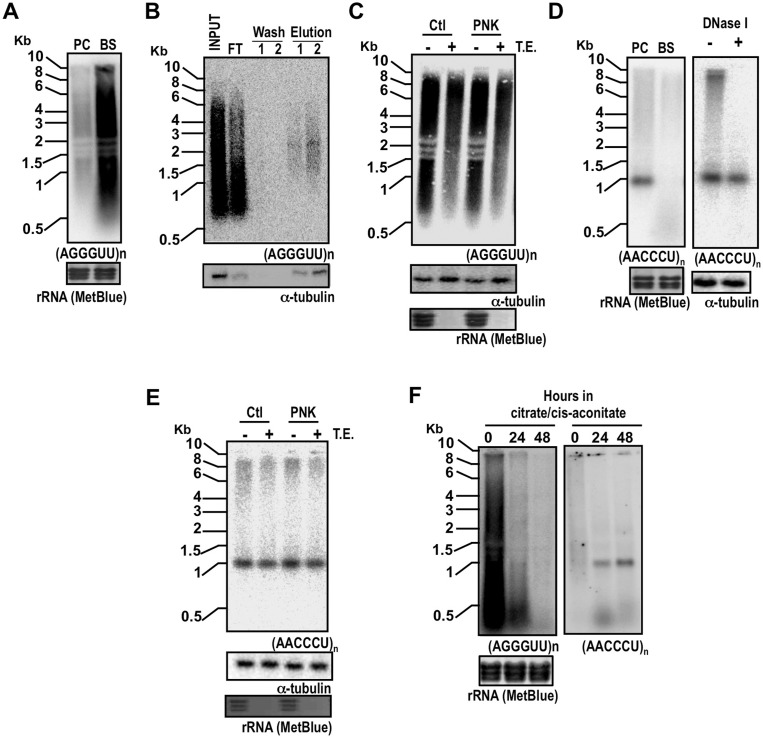
: transcription of UUAGGGn-containing RNAs (TERRA) from *Trypanosoma brucei* telomeres. (A) Total RNA (~15 µg) from procyclic form (PC) (29.13 cell line) and bloodstream form (BS) (SM cell line) of *T. brucei* were subjected to Northern blot analysis as in Fig. 1A; rRNA (MetBlue) refers to visualisation of rRNA after transfer to the membrane and staining with methylene blue and serves as a loading control. (B) Total RNA from BS form of *T. brucei* was fractionated on an oligo(dT) resin (Oligotex mRNA Mini Kit, QIAGEN); equivalent volumes from Input, flow through (FT), washes and eluted material were analysed by Northern blot analysis; the material was probed for a-tubulin as a positive control for the binding of poly(A)+ RNA. (C) Total RNA (~15 µg) from *T. brucei* BS forms was left untreated (Ctl) or subjected to PNK treatment before incubation with TE enzyme; the membrane was probed for a-tubulin as a control for 5’CAP-containing RNA. rRNA(MetBlue) serves as control for 5’ mono-phosphorylated RNAs. (D) Left panel: Northern blot analysis of total RNA (~15 µg) from *T. brucei* PC and BS forms; rRNA (MetBlue) refers to visualisation of rRNA after transfer to the membrane and staining with methylene blue and serves as a loading control; right panel, total RNA (~15 µg) from *T. brucei* PC forms was left untreated or treated with DNase I and then subject to Northern blot analysis; the same membrane was probed for a-tubulin as a loading control; DNaseI activity was confirmed using plasmid DNA as substrate, under the same reaction conditions. (E) Total RNA (~15 µg) from *T. brucei* PC forms was treated as in C and then subjected to Northern blot analysis. (F) *T. brucei* BS form cells were incubated in medium containing citrate/cis-aconitate to induce the differentiation into PC forms as previously described (Ziegelbauer et al. 1990); total RNA was extracted at the indicated time points after the induction of differentiation and subjected to Northern blot analysis.

Similar to *L. braziliensis*, RT-PCR analysis indicated that TERRA is also polyadenylated in *T. brucei* BS forms (Fig. 1C). Also, fractionation of total RNA in oligo(dT) resin indicated that, as for *L. braziliensis*, at least a subset of the total TERRA transcripts is polyadenylated in BS *T. brucei* (Fig. 2B). This is comparable to *Leishmania* TERRA and somewhat lower than the polyadenylated telomere transcripts detect in the PC forms of the parasite, as previously reported (Rudenko & Van der Ploeg 1989), which could be due to differences in parasite cell lines. We also analysed the susceptibility of TERRA from *T. brucei* BS forms to TE treatment. As shown in Fig. 2C, a considerable proportion of TERRA was susceptible to TE treatment, indicating that these molecules possess a mono-phosphorylated 5’-end structure. Prior treatment of RNA with PNK did not alter the susceptibility of TERRA to TE suggesting that the TE-resistant fraction of TERRA does not have a free 5’-hydroxyl end. It remains to be determined whether TERRA in BS forms of *T. brucei* possess a di or tri-phosphorylated or a CAP structure at the 5’ end.

Surprisingly, when we probed the *T. brucei* RNA samples for antisense transcripts (CCCUAA)_n_ we detected a distinct transcript of ~1,0 Kb in PC forms that was absent in BS forms (Fig. 2D). This transcript was resistant to DNase I treatment, excluding that it was due to DNA contamination. Whether this RNA entity represents a *bona fide* anti-sense transcript from a single telomere in PC forms or constitutes a developmentally regulated transcript bearing homology to the telomere probe remains to be elucidated. Treatment of total RNA with TE did not affect the levels of the (CCCUAA)_n_-containing transcript (Fig. 2E). In addition, PNK pre-treatment did not affect the susceptibility of the (CCCUAA)_n_ transcript to TE incubation. This data suggested that this transcript neither has 5’-mono-phosphorylated end nor a free 5’-hydroxyl group. Considering that the BS and PC forms used in the experiment shown in Fig. 2A were non-isogenic strains, we decided to investigate TERRA levels in BS and PC derived from the same *T. brucei* cell line. For this, we compared TERRA levels before and after the induced differentiation from BS to PC forms in the single-marker (SM) cell line. As shown in Fig. 2F, TERRA levels were drastically reduced in newly differentiated PC forms when compared to undifferentiated BS forms. Finally, the anti-sense TERRA transcript was detected only after the differentiation of BS to PC forms.

Our findings suggest that, similar to other eukaryotes, TERRA is a distinct transcript class in trypanosomatids. The initial characterisation we present here suggested that TERRA levels seem to be controlled throughout the life cycle of *L. major* and *T. brucei.* Further studies are required to dissect the underlying biological mechanisms leading to the differential processing of 5’ end of TERRA of *L. braziliensis* relative to *T. brucei*. Perhaps this is the result of the different RNA polymerases involved in TERRA biogenesis in the two parasites. Also, the role of TERRA in the maintenance and functioning of the trypanosomatid genome and of the antisense telomere-sequence containing RNA expressed in procyclic forms of *T. brucei* should also be the subject of further investigation.
